# Beyond words: operationalizing inclusive language in Australian cervical screening health promotion policy

**DOI:** 10.1093/heapro/daaf058

**Published:** 2025-05-20

**Authors:** Kerryn Drysdale, Nicola S Creagh, Claire Nightingale, Lisa J Whop, Angela Kelly-Hanku

**Affiliations:** Centre for Social Research in Health, UNSW, Warrane (Sydney), Australia; The Kirby Institute, UNSW, Warrane (Sydney), Australia; Melbourne School of Population and Global Health, The University of Melbourne, Naarm (Melbourne), Australia; Melbourne School of Population and Global Health, The University of Melbourne, Naarm (Melbourne), Australia; Yardhura Walani, National Centre for Aboriginal and Torres Strait Islander Wellbeing Research, The Australian National University, Ngambri (Canberra), Australia; The Kirby Institute, UNSW, Warrane (Sydney), Australia

**Keywords:** health equity, health promotion, LGBTIQA+, gender and sexuality diverse, National Cervical Screening Program, policy implementation, women’s health, gender inclusion

## Abstract

Health equity is a fundamental concern within the broader health promotion aim of creating equal opportunities for health and bringing health differentials down to the lowest level possible. Cervical screening is just one example of a preventative health program where a health promotion lens is required to address entrenched health inequities. We draw on theorizations of policy ecologies to provide a framework for better understanding the processes involved in operationalizing policy with greater inclusivity in language in health promotion. Twenty-eight semi-structured interviews were conducted with 29 key informants between April and October 2022 to explore the operationalization of inclusive language in health promotion in the context of a national program to promote cervical screening to currently underscreening communities in Australia. Four thematic categories emphasize the balance required between demands and domains: (i) the need for clinical guidelines and flexibility in their translation and interpretation; (ii) organizational mandates, clinical practice, and patient-centred care; (iii) socio-cultural norms, behaviours, and attitudes amid politicized/ing milieus; and (iv) community preferences and the need for medical accuracy. As such, we identified how the operationalization of inclusive language in policy is influenced by and influences other domains where cervical screening is promoted. These findings hold wider implications for how the historical legacies of and contemporary need for ‘women’s health’ can be maintained and respected amid demands for greater gender inclusion. At the same time, the failure to trace diverse and diffuse modes and contexts of operationalization may (re)produce health inequities in practice if left unexamined.

Contribution to Health Promotion

- Effective and resonant inclusive language in health promotion is a health equity issue.
- Cervical screening in Australia has increasingly promoted the use of inclusive language in health promotion policy.
- The needs and preferences of currently underscreening populations need consideration to ensure that they engage with cervical screening.
- Policy ecologies provide a framework for understanding the downstream operationalization of health promotion policy.
- Inclusive language is operationalized within and across clinical, organizational, community, socio-cultural and political domains, not just in policy mandates alone.
- Findings suggest how greater inclusivity can be adapted within the medical domain of ‘women’s health’.

Health equity is firmly centred within the foundational aims of health promotion. As a core principle in responding to entrenched health inequities, health promotion seeks to move beyond a focus on individual behaviour to address the social, environmental, commercial, and structural determinants of health that influence health behaviours and outcomes, including gender, race, sexuality, (dis)ability, and so on (Braveman 2006, Mouy and Barr 2006, Baum et al. 2009, Corbin 2016, Ndumbe-Eyoh 2020, Newman et al. 2021, Björklund and Lindroth 2024, Ertl et al. 2024). A concomitant focus on equity is therefore concerned with creating equal opportunities for health so as to bring health differentials down to the lowest level possible (Whitehead 1991, see also Braveman 2006). An important aspect of health promotion and preventative health programs is to assess the needs of disparate groups who may be experiencing health disparities, and tailor policies, programs, and promotional activities to address differences in health risk factors.

Cervical screening is just one example of a preventative health program where a broad health promotion lens is required to address entrenched inequities globally, including in high-income countries such as Australia. Through prevention, including cervical screening, cervical cancer is almost entirely preventable (Brotherton et al. 2025). Despite this, cervical cancer remains a cancer of inequity, with large disparities in access to preventative programs resulting in unnecessary suffering of women and people with a cervix. In 2020, the World Health Organization released a landmark strategy, which called on all countries to reach a threshold of < 4 cases per 100 000 in order to eliminate cervical cancer as a public health concern (World Health Organization 2020). Achieving high population-level cervical screening rates is one of the three core components of this strategy. While Australia may be one of the first countries to achieve the elimination threshold for cervical cancer at a whole-of-population level (Hall et al. 2019), this may not be achieved for some populations who face systematic barriers to healthcare, including (but not limited to) gender and sexuality diverse people (Gibson et al. 2021, Kerr et al. 2022, Rivers et al. 2024, Ellis 2025).

This article presents findings from a targeted qualitative study of the role and impact of, and decision-making processes for, inclusive language in cervical screening policy, promotion, and delivery in the context of the National Cervical Screening Program (NCSP) in Australia. We sought to identify the processes, including enablers and barriers, for how health promotional policy around language in domains traditionally associated with gendered health, such as cervical screening, is or can be successfully operationalized. Here, we seek to further understand if and how inclusive language is adopted (or adapted) by key stakeholders, the relationship between inclusive language in health policy and its downstream operationalization in health promotion in clinical, organizational, community, socio-cultural, and political domains, and how the operationalization of inclusive language in policy is influenced by and influences such other domains where cervical screening is promoted.

## BACKGROUND

### ‘Women’s health’, cervical screening, and the ongoing imperative for inclusivity

As a distinctive biomedical field, ‘women’s health’ emerged as a result of the activism and advocacy associated with second wave of feminism in the 1960s and 1970s in the Global North (Nichols 2000). Women’s demands for control over their reproductive lives were underpinned by two interrelated rights-based political discourses: recognition of their rights to reproductive choice, and calls for an end to sexism, patriarchy, and paternalism in healthcare systems. Professional medical bodies responded by establishing a new medical speciality of ‘women’s health’. The incremental development of ‘women’s health’ thus emerged from the recognition that, historically and consistently across a broad range of health specialities, data have been collected from men and inappropriately generalized to women (Institute of Medicine 2001); in effect denying consideration of women’s differences from men (Mauvais-Jarvis et al. 2020). In calling for a ‘global agenda for women’s health’ (Doyal 1996), the distinctive domain of ‘women’s health’ now includes many aspects of healthcare, including cervical screening.

While evidence that speaks to topics that fall under the broad consideration of the health and well-being of women is plentiful, the term ‘women’s health’ itself is rarely subject to scrutiny (Nichols 2000, Inhorn 2006, Hankivsky 2012). While careful not to dismiss this history of activism and advocacy, the ambiguity of what might appear as taken-for-granted definitions continues to unsettle the universality and applicability implied in the term ‘women’s health’ (Bush 2000, Sundstrom et al. 2019, Gibson et al. 2021). For instance, the term ‘woman’ might not be appropriate for individuals who require specialist clinical services related to their anatomy but who may not identify as women, and some of the services offered under the domain of ‘women’s health’ may not be applicable to all women (cis or trans). ‘Women’s health’, then, can be exclusionary by default if health services are promoted for only (cisgender) women, which can create tension if ‘women’s health’ is conceived solely in terms of difference *from* (cisgender) men. As such, any predominant emphasis—either explicit or implied—on heteronormative and cisnormative subjects in healthcare policy and provision risks the systemic neglect of gender and sexuality diverse people (Moseson et al. 2020, Morrison et al. 2021, Rioux et al. 2022), if structured by a binary differentiation between ‘women’s’ and ‘men’s’ health (Hankivsky 2012). Accordingly, the partiality of definition related to gendered subjects has prompted moves towards gender inclusivity that acknowledges experiences of, in particular, trans men and non-binary people within the domain of women’s health (Stroumsa and Wu 2018, Sundstrom et al. 2019, Moseson et al. 2020, Gibson et al. 2021). We find that contemporary efforts towards inclusive language are now concerned with the principles of inclusion of all genders (rather than the previous focus on difference from one default gender, i.e. the universal male subject).

A key domain where shifts in language to make ‘women’s health’ more inclusive is currently being negotiated is within Australia’s cervical cancer prevention efforts; specifically, its longstanding National Cervical Screening Program (NCSP) established in 1991. For example, within this program, moves towards gender inclusivity can be seen in the introduction of a gender-neutral school-based HPV vaccination program (i.e. for all young people, not just girls) from 2013. The imperative towards health equity is now also evident in efforts to increase cervical screening among priority populations in Australia (identified as Aboriginal and/or Torres Strait Islander people, culturally and linguistically diverse people, gender and sexuality diverse people, people with innate variations of sex characteristics, people with disability, and people living in rural and remote areas) (see Australian Centre for the Prevention of Cervical Cancer 2023). To increase the uptake of cervical screening, language around who is eligible for cervical screening became a priority for consideration, centred around the broad recognition that the language used in health promotion can either emphasize or suppress socio-cultural aspects of healthcare that then influence its uptake (Fry 2020).

Australia’s National Strategy for the Elimination of Cervical Cancer in Australia (the Strategy), launched in November 2023, uses the words ‘eligible people’ and ‘women and people with a cervix’ (and includes caveats when the terms ‘women’ and ‘girls’ were used exclusively when citing other sources; see Australian Centre for the Prevention of Cervical Cancer 2023). Read in terms of inclusivity, the language of the strategy makes it clear that cervical screening does not solely reside within ‘women’s health’, nor does it recommend only targeting cisgender women as its intended audience for health promotion. Indeed, the strategy embeds diversity as an equitable health outcome for all people with a cervix and includes recommended strategies that emphasize the importance of health promotion for gender and sexuality diverse people (among strategies for other identified underscreening populations).

### Policy ecologies and processes of operationalizing inclusive language

Addressing the systemic causes of health inequity requires intentional and multipronged actions, including at the policy level, population scale, and within specific healthcare domains. As such, the operationalization of health policy is equally a consideration in achieving health equity (Brownson et al. 2021). While inclusive language in health policy may offer stakeholders within the NCSP a rationale and instruction for its application within clinical and organizational settings, how these instructions are influenced by broader settings, such as community, political, and socio-cultural domains, is equally important to consider. Accordingly, we draw on theorizations of policy ecologies to provide a framework for better understanding the processes involved in the downstream operationalization of policy with greater inclusivity in language in health promotion.

As formulated in the context of evidence-based practices in mental health policy (Raghavan et al. 2008, Wortham et al. 2023), a policy ecology of implementation approach warrants consideration of policy ‘activities’, which the authors identify as needing to take place in and in respect to organizational contexts, regulatory and funding environments, political milieus, and socio-cultural norms: ‘policymakers need to align the effects of policy action across all of these contexts in order to produce sustained, systemwide uptake’ (p. s3). Understanding the influence of diverse contexts, as Bradley and colleagues (2024) also note, goes beyond understanding contexts as a mere backdrop in which health interventions and health promotional programs are implemented; contexts ‘possess an active and dynamic role exerted through interaction, influence, modification, facilitation, and constraint’ (p. 4). Accordingly, how inclusive language in health policy is operationalized becomes a question of adoption and adaption contingent upon the context and the intended purpose, including diverse settings. How a policy moves from one domain into another—and therefore into an actionable set of activities—is a critical consideration.

This consideration may also require attention to broader policy processes and impacts to identify how policies extend beyond their ‘artefactual’ guise (referring to written statements of intent, guidelines, restrictions or dictates as an instrument of government alone) (Lea 2020). As Lea (2024) further demonstrates, policies’ depth and breadth also include recognition of their ambient (that is, policy’s elusive role in shaping everyday conditions where policies are tacitly embedded in various contexts and practices) and hauntological (that is, how past policies’ reverberations shape prior social configurations so as they appear predetermined and inevitable) modalities. This means that single policy interventions do not occur in isolation but are shaped by past and present policy instantiations. The often-variable ways in which specialist healthcare is consolidated under umbrella domains, such as ‘women’s health’, as well as the historical legacies of feminist activism in the development of such domains, may facilitate a greater understanding of health promotion policy interdependencies, affects, and implications when operationalized.

Our intention in presenting these more capacious conceptualizations of policy and the expansive relationships between policy and health promotion is not to directly apply these to the development of inclusive language in cervical screening policy and evaluate its implementation. Rather, using this framework allows us to see the wider contexts and processes in which policy can (or should) be operationalized. Specifically, we were interested in how inclusive language in health policy is adopted (and adapted) within different clinical, organizational, community, socio-cultural, and political settings beyond a mandate for use.

## METHODS

Potential key informants were identified through the research team’s professional networks and participant snowball sampling. Invitations relied on purposive sampling processes to ensure a mix of metropolitan, regional, rural, and remote locations, in professional roles such as generalist clinicians (nurses and doctors) in women’s health (including obstetrics/gynaecology), cancer prevention (both government and non-government organizations), as well within organizations serving specific populations (Aboriginal and/or Torres Strait Islander peoples, gender and/or sexuality diverse people, people with a disability, rurally based or geographically isolated people, and some culturally and linguistically diverse communities). Written or verbal consent was collected at the outset of each interview. No incentive was offered to participate in an interview.

Interviews were conducted by the first two authors, and the interview schedule can be found in the Supplementary File. Interviews were recorded and transcribed verbatim. Transcripts were thematically coded using qualitative software (QSR NVivo version 12) using inductive thematic analysis (Terry et al. 2017). Provisional themes were refined through a process of open thematic mapping and repeated re-examination of the data and subsequently refined themes were retested against the data.

Two streams for analysis were ultimately devised through the process of deep immersion in the data. The first, the role and function of inclusive language in cervical screening policy, is described elsewhere (Drysdale et al. 2024). The second, how inclusive language in cervical screening policy is operationalized in health promotion across clinical, organizational, community, political, and socio-cultural settings, is the subject of this article. Accordingly, this article builds on prior findings to explore in more detail how inclusive language policy is adopted or adapted in the context of the NCSP. Data used for this article were primarily taken from coding nodes on the processes of language changes in different contexts (clinical, organizational, community, political, and socio-cultural), the enablers and barriers to language change (policy levers, organizational acceptance, community expectations, social norms, and political appetite), and the perceptions on how to ‘do’ inclusive language in the promotion of cervical screening targeting currently underscreening populations. To protect the anonymity of key informants, broad categories are used to describe the domain of expertise in the sample (cancer policy, health promotion, healthcare delivery, and population group advocacy). Ethical approval was provided by the UNSW Human Research Ethics Committee (UNSW HC220031).

## RESULTS

A total of 28 semi-structured interviews were conducted with 29 people (one interview was conducted with two key informants from the same organization) between April and October 2022, each interview lasting between 31 and 56 min in length (median 46 min) (see Table 1). Key informants provided diverse professional perspectives on if and how inclusive language in health policy was operationalized in health promotion and education, and healthcare delivery with respect to cervical screening. The phrasing of ‘women and people with a cervix’ was used throughout the interview to reflect the language of policy documents, with key informants asked if and how this phrasing was operationalized in their work.

**Table 1.** Key informants’ demographic characteristics

Characteristic	Total ( *n* = 29)
**Domain of professional expertise** Cancer policy Health promotion Healthcare delivery Population group advocacy	8 (28%) 5 (17%) 9 (31%) 7 (24%)
**Age** 20–29 years 30–39 years 40–49 years 50–59 years 60 + years	3 (10%) 4 (14%) 6 (21%) 6 (21%) 10 (34%)
**Gender** Woman (cis and trans) Man (cis and trans) Non-binary	25 (86%) 2 (7%) 2 (7%)
**Sexuality** Heterosexual Gay/Lesbian Queer/Pansexual Not specified/preferred not to say	15 (52%) 8 (28%) 4 (14%) 2 (7%)
**Indigenous status** Aboriginal and/or Torres Strait Islander Not Aboriginal or Torres Strait Islander	3 (10%) 26 (90%)
**Country of birth** Australia Other Not specified/preferred not to say	21 (72%) 7 (24%) 1 (3%)
**State of residence** New South Wales Victoria Tasmania Western Australia Northern Territory Australian Capital Territory Queensland	10 (34%) 10 (34%) 3 (10%) 2 (7%) 2 (7%) 1 (3%) 1 (3%)
**Rurality of residence** Metropolitan Regional Rural Remote	18 (62%) 5 (17%) 3 (10%) 3 (10%)

Our analysis was focused on the processes of adoption and adaption of inclusive language amid wider policy ecologies. Four thematic categories were produced through this analysis, each implying an intersectional and intersectoral relationship between the various contexts in which inclusive language policy was operationalized.

### Balancing the need for clinical guidelines and flexibility in translation and interpretation

All key informants valued the importance of language as fundamental to effective health promotion; that is, to ensure that people engage with healthcare systems—and subsequently receive appropriate healthcare. Effective clinical delivery, then, required recognition of the broader context in which health care is delivered, including health promotion strategies sponsored, promoted, and/or provided under the domain of ‘women’s health’:

Oh gosh! Language is crucial. Like it’s vital because it can stop people from accessing inclusive healthcare at that front door or at that phone call, at that referral moment. […] and I’ve heard of stories of people who don’t follow through with healthcare because it’s too hard without that inclusive language. (Interview 11—population group advocacy)

As such, inclusive language was seen by this key informant to go beyond cervical screening, and that it should be embedded throughout healthcare systems. Yet, effective and resonant health promotion requires, at the very least, clinical guidelines need to be inclusive as a starting point for the wider aim of health equity:

But I do think at the very least, all guidelines need to recognise diversities. (Interview 6—cancer policy)

The need to embed inclusive language was also perceived among other key stakeholders, whether it has been mandated through top-down health policy or is a result of employee-directed preferences. That is, despite the form the imperative took, it was up to people themselves to operationalize inclusive language at an organizational level:

So, those of us who do a lot of cervical screening and breast exam and other gendered examinations, you know, we have increasingly been more careful with our language when we talk to each other. (Interview 9—healthcare delivery)

At the same time, there was a recognized need among key informants for flexibility in the interactions between healthcare providers and patients:

I mean, policy is made for the caregivers [cervical screen providers] anyway. It’s not necessarily made for the consumer, so I don’t think it matters that much, so long as the people that kind of conveying it down to the client actually can do that sensibly and appropriately. I mean, most policies aren’t written for people. (Interview 28—health promotion)

In this way, key informants saw that inclusive language in health policy was directed to them, as healthcare providers, in ways that offer guidance to its operationalization, but the specific forms that this took within the clinical encounter were up to the healthcare provider to translate in respectful, culturally appropriate, and resonant ways.

It is not surprising that throughout the interviews that there were reasonable preferences for language among the key informants, who are often acutely aware of how language impacts on the people and the communities they serve. The benefit of the flexibility in healthcare providers is in determining which mode of communication is best for the priority population to whom they are promoting cervical screening.

### The balance between organizational mandates, clinical practice, and patient-centred care

It is one thing to mandate inclusive language through health policy, but another thing to ensure its successful operationalization in diverse organizational settings. For health promotion that falls under the traditional domain of ‘women’s health’, such as cervical screening, a significant obstacle in policy operationalization was healthcare providers’ appetite for change and their willingness to adopt (and adapt) from previously gendered (and therefore non-inclusive) to non-gendered (and therefore inclusive) language—whether this was mandated through clinical guidelines or not. Key informants were aware of the potential (and perhaps even inevitable) misalignment in the flow of language between policy and its downstream operationalization:

Well, I think it’s a spectrum. So, at the one-to-one level with the patient, you’ve got to be as flexible as you can be as possible. So, you’re accommodating their needs. Then, at community level, it’s a little less flexible, you know. Then, at the policy level, its big picture. (Interview 9—healthcare delivery)

One key challenge, as one key informant put it, is the need to actively work to challenge assumptions made about what anatomy certain bodies have based on which (gendered) healthcare service that they required. While people may access cervical screening through services banded under medical disciplines (which may be explicitly or implicitly organized under the domain of ‘women’s health’), key informants recognized that such services are widely perceived to be (already) gendered:

And this is the thing, that it’s not about bodies, it’s about bodies that are moving into gendered spaces. (Interview 12—population group advocacy)

Put simply, if a person is to engage in cervical screening, they often do so with the recognition that this service is provided under the auspice of ‘women’s health’. However, this does not reduce the burden on healthcare providers to ensure that this clinical service is appropriate for the person who requests it:

Look, I think that there’s nothing wrong with women’s health services being delivered for women, provided they’re delivered for trans women too. […] but I think then that [women’s] health services need to really kind of be enabled around providing, you know, particular services and screening to priority populations who perhaps are unserved or may not experience what they need to experience in terms of the highest attainable level of care. (Interview 11—population group advocacy)

Likewise, community conventions—and the way that cultural traditions impact on health promotion—need to be considered in determining the best language to be used to target underscreening populations (and indeed, what inclusive language even looks like in differing contexts):

[Inclusive language] is very distinct, you know. Inclusive is very much about women and if you talk about women’s business, it’s only for women, you know? So, I think they would find it very hard. If you were talking to say, a man with a cervix, like a transgender male, that wouldn’t fit their scenario, you know, that person on the outside looks like a male, so it wouldn’t fit. (Interview 28—health promotion)

As this representative quote on promoting cervical screening to—in this example, Aboriginal and/or Torres Strait Islander peoples—suggests, health messaging needs to align with a person’s worldview. Accordingly, key informants recognized that, at the clinical practice level, they are mandated with the need to ensure that they are engaging with their patient in the best possible way to ensure screening uptake:

I suppose if you’re doing some education for clients, then I would probably make it less formal, a bit more fun, a bit more trying to … trying to make it applicable to them, wherever they come from, cultural or background or whatever. (Interview 7—healthcare delivery)

Careful wording needs to be selected to ensure that health promotion messaging lands, especially for underscreening populations such as Aboriginal and/or Torres Strait Islander peoples or culturally and linguistically diverse people. Key informants spoke to the need to balance the aim of being inclusive with the risk of incomprehension, and that this requires organizational capacity to have ‘awkward’ conversations to clarify the intention behind inclusive language in order to ensure socio-cultural acceptance:

You know, if that means someone having to have an awkward conversation about who are the people that have got cervix, well, ‘have you considered transgender people? That’s great’. [These are] little opportunities that we believe [are worth] the risks that we take, we just go for it. So, I think we should still do that, but we need to be prepared for the questions that will come. And in time those questions will stop. But, you know, we are talking about [culturally and linguistically diverse] populations that have strong religious beliefs as well. (Interview 1—cancer policy)

As indicated by this quote, recognizing the influence of socio-cultural factors into other domains, whether it be within clinical or organizational contexts, requires tailoring to different audiences that goes beyond the mandate for inclusive language in health policy.

Key informants expressed strong preferences for a person-centred approach to language that was based on their acknowledgement that there were significant challenges in finding terminology that accurately reflects the needs and preferences of a diverse range of communities. Many key informants spoke to the need to develop strategies to prioritize person-centred language to minimize the need for (de)gendered terms:

With our resources in our communications, we also try to move towards instead of using the sort of the third person terminology. For our consumer-facing resources wherever possible, we use ‘you’ […] So moving it into the second person kind of obviates the need for having to continually use this category of ‘women and people with a cervix’. (Interview 4—health promotion)

In effect, strategies such as these ameliorate the need to identify a gendered subject to whom cervical screening is promoted. In practice, this might require healthcare providers to ask questions regarding the body parts of the individual who presents to them:

So in clinical practice, we need to make sure that clinicians are undertaking what is called - I don’t really love the name, still yet to find a better name - but it’s called an ‘organ inventory’, which is where part of the intake process, part of the relationship building process about ‘what body parts do you actually have’, and then making a note, and then providing clinical care based on that. Not based on anything called male or female. (Interview 12—population group advocacy)

Abstracted from the imperative towards inclusivity, specific inclusive language strategies that promote the use of de-gendered language, either by removing the subject from the description of the anatomy, practice, or medical issue or by using first- or second-person language, were also seen by key informants to be best practice that caters to a trauma-informed approach to health promotion:

One of the things that’s fairly common between some of those populations is a higher than normal or higher than average experience of trauma. So, our approach is highly trauma informed, whether it be messaging or service delivery, so that is a key kind of thing that I think works for us and a good trauma informed care is better for everybody in my view, not just the person with the trauma. (Interview 14—population group advocacy)

A de-gendered linguistic strategy, underpinned by a trauma-informed approach, then, was seen by key informants to be of benefit to women and gender-diverse people alike. Yet, the potential influence of adopters of inclusive language in policy or organizational contexts are still reliant on their own commitment to be catalysts for change within their realm of influence. The issue remains as to the extent to which others choose or not to adopt an inclusive language approach in their work. This suggests that there are further contexts for consideration.

### The balance between socio-cultural norms, behaviours, and attitudes amid politicized/ing milieus

The phrase ‘women and people with a cervix’ was generally considered by key informants to be more politically acceptable than the term ‘people with a cervix’ alone (an exception to this was two key informants who queried the need for the additive ‘and people with a cervix’ in addition to the gendered subject ‘women’ in reference to who was eligible for cervical screening, and one person who preferred the term ‘people with a cervix’ without the additive ‘women’). The retention of ‘women’ was seen by many key informants to reflect the historical legacies and ongoing importance of women’s health advocacy that had traditionally seen ‘women’s health’ as minority population and/or service in health more generally. This also includes recognition by key informants that this domain continues to hold resonance among organizations serving women as a population recognized to have different or unique health related needs and/or preferences. But for many underscreening populations, key informants noted that inclusivity might be better framed as a health literacy issue:

I think the easiest thing to say is ‘people with a cervix’ because it’s to the point and I think it’s quite widely understood. But like I said before, there is not enough level of education to assume that everyone understands where their cervix is or what it is. (Interview 16—health promotion)

At the same time, key informants were keenly aware of the larger debates going on, and were cognizant of the politicized nature of inclusive language:

One of the challenges that I’ve learned the hard way is that it is difficult to make one size fit all, and so some of the criticisms that we’ve received has been by referring to people rather than women. You know, the vast majority of the population who are likely to develop cervical cancer view themselves as women and if they are not terribly sophisticated, do not understand inclusive language [then] it is a constant tension and battle trying to navigate that very, very razor-thin pathway of not offending anybody. And quite frankly, I think it’s impossible. (Interview 10—cancer policy)

The care not to cause offence was clearly perceived by key informants to be an inevitable part of the political milieu in which health services are promoted—and may not be entirely unavoidable. While it is not surprising that linguistic preferences differed between priority populations, this above quote demonstrates the care with which language was selected in the context of an acknowledgement of the inevitability that healthcare providers may never get this right all the time. Nonetheless, all key informants agreed that language used to promote cervical screening needs to be culturally relevant (and safe) for people when engaging with healthcare delivery:

I think with health promotion, I think there is that segmenting of the market and, you know, you are wanting to appeal to particular groups […] Inclusivity, inclusive language, I mean, I think we have to use language that works for them and choose that carefully. (Interview 6—cancer policy)

At the same time, the operationalization of inclusive language in clinical encounters relies on clinicians being familiar with (and indeed, trusted by) the communities they promote screening to:

Well, you must remember that, you know, clinicians that work in these areas generally are very skilled at being able to read guidelines but know how to apply it in their own communities and how to use it. So, you could have the policy, you know, in a politically correct language but then, say, here’s some suggestions of how you could use it in your communities. (Interview 26—healthcare delivery)

An organization’s visible connection to community, and the role of peers in making that connection explicit, was seen as part of an organization’s mandate to inclusive language, often relying on longstanding ties between organization, community, and socio-cultural contexts:

And with things like AMSs [Aboriginal Medical Services], that’s great because you know you have got an Aboriginal message, you have got an Aboriginal service […]. But this notion that community trust, community language, you know, service navigation for health, […] it doesn’t occur through, you know, in any other formalised way, so that trusted message is really key. (Interview 14—population group advocacy)

Nevertheless, there were best practice models put forth for the processes of organizational decision-making around inclusive language, often including regular review and building co-design and consultation with relevant communities into the process:

So, consultation with the community and co-design. So, not just consultation, not just going and having a yarn and thinking, ‘Oh, now we know it all. We will go off and create the policies’. Co-design along all the steps of the way and then when, you know, those policies are implemented, review by that community, ongoing review as well because language changes across time […] So, it needs that constant review along the way. It can’t just be created by someone who thinks they know it all. (Interview 11—population group advocacy)

Indeed, key informants emphasized the importance of community input (and leadership) into the language that was normalized in organizational contexts in which cervical screening was promoted to currently underscreening populations:

The other thing is that you really need community leadership, it can’t come top down from government and people, and the same message for everybody and expect it to work. So, peers, community elders, respected organisations, they really need to be involved in the messaging, but also if you can, the service delivery aspect of it as well. (Interview 14—population group advocacy)

This quote speaks to key informants’ perception of the intertwined and imbricated roles of community leadership and consumer advocacy for driving change at multiple levels of potential uptake.

Accordingly, the strategies advanced by key informants to minimize the exclusion of people from the domain of ‘women’s health’ was based on the recognition that ever-increasing categorization of people to medical specialities was never going to be inclusive to all people who may require specialist clinical services:

We’ve spent a lot of time putting people in boxes, and now we’re trying to get people out of boxes and on the whole in terms of healthcare, I think that we need to acknowledge that we’ve been trying to make health care more individual focused and less sort of, you know, generalised anyway, so I think that’s part and parcel with that movement of trying to make things more patient specific and focused. (Interview 19—healthcare delivery)

There was recognition of the significant investment in ‘women’s health’ for cervical screening, as this was considered the most appropriate domain under which such health promotional policies should be developed and implemented (as opposed to, for example, within sexual health sector more broadly, as this was seen to be culturally inappropriate for some culturally and linguistically diverse communities). As such, key informants spoke to the need for adaptation, both in terms of their own practice and in terms of how the domain of ‘women’s health’ is adapted/can be adaptable towards greater inclusivity.

### Balancing community preferences with the need for medical accuracy

Operationalizing inclusive language in cervical screening policy into clinical and community domains requires the careful balancing of community preferences for the language used in respect to body parts and cervical screening with the need for clinically accurate terms to convey with medical information. Health promotion needs to be medically accurate to ensure that the right service is provided to the people for whom it is intended—and healthcare providers were seen as key conduits through which inclusive language was adopted and adapted to community settings. For example, overly formal or medicalized language may not be directly translatable to lay audiences who do not have specific medical literacy:

I think one of the biggest issues as far as language is concerned in ensuring that we are speaking in a way that is comprehensible to the vast majority of people. And I think there has always been a tendency to over-medicalise and formalise language, which means it is too dense and incomprehensible to many people. Interestingly, still today, many people confuse ovaries with the cervix. (Interview 10—cancer policy)

On the other hand, community preferences for language that speaks to their lived experience may render medical terminology irrelevant or inappropriate. This was evident in views from key informants who worked with populations for whom English was not their first language:

And the difference with very remote as well because, you know, for people where I mainly work, English is their third, fourth or fifth language. So, when it comes to using language, the other thing is there’s a very simplified English way of speaking. (Interview 26—healthcare delivery)

This quote reflects that differences within each priority population may also have distinctive preferences for the language used when referring to medical procedures. In practice, this means that healthcare providers who promote cervical screening to their patients also had their own processes of adaptation, holding both medical precision and community preferences in balance depending on who they were talking to and in which contexts:

I guess, you’ve chatted to someone for five minutes before you get into that aspect. If I’m in [regional city] and I’m talking to many, you know, more highly educated that I am, you know, there’s no way I would not use the word ‘vagina’, ‘vulva’, things like that. Whereas if I’m in [remote township], it’s a whole another world because they know five other languages before English and so you have to choose a word. I think they would understand ‘vagina’ if you use that word, but I think they would find it a little bit more taboo than using their local word. (Interview 27—healthcare delivery)

Given the complexity of different linguistic preferences, the promotion of cervical screening to currently underscreening populations often went beyond words. Key informants often used multiple modes of communication when explaining the need for cervical screening, including the use of visuals and anatomic models:

So certainly, from a clinical perspective it’s the responsibility of the clinician to make sure they are using appropriate terminology, appropriate explanations, that they have, you know? A lot of them will have gynaecological model on hand or diagrams. I actually think is very, very important that people understand what the procedure is, especially something like cervical screening. (Interview 2—cancer policy)

Alternative modes of communication were perceived to increase the potential for understanding where on the patient’s body the screening would take place, and what screening involves. Importantly, key informants framed such alternative modes of communication in terms of effective health literacy, not solely in terms of customization to priority populations:

I would say whenever we’re doing education or getting consent or anything, we use pictures, models. So, there was a lovely little model of a uterus and cervix and all that. […] And also, the other really great way of doing education is doing stories, telling a story about someone, like storytelling. (Interview 26—healthcare delivery)

Such enhanced communication tools were also seen by key informants to be easily available and accessible in their clinical encounters with patients:

I have access to the internet in most places that I work in, or my smartphone. So, I will always bring up appropriate diagrammatic representations and, with their permission, I will explain things with those as well. (Interview 18—healthcare delivery)

While there may be a tension between the gendered nature of ‘women’s health’ under which cervical screening is promoted and provided and the mandate for greater (gender) inclusivity in health policy, the adaptability within and across various domains associated with cervical screening health policy means that this tension rarely surfaces in practice—and if it does, other modes of communication sidestep these tensions.

## DISCUSSION

In this article, we explored the role and impact of, and decision-making processes for, inclusive language in cervical screening policy, promotion, and delivery in the context of the National Cervical Screening Program (NCSP) in Australia. Through this targeted, qualitative study, we identified diverse policy ‘activities’ that were required within and across various clinical, organizational, community, socio-cultural, and political domains where cervical screening is promoted.

Inclusive language in policy, including those translated to clinical practice guidelines, is just one step in driving adoption and adaption in health promotion practices. Indeed, the intended purpose of inclusivity within policy may or may not reach those who would most benefit from such language because policy requires actors to operationalize it (see, for example, Austad et al. 2016, Eslava-Schmalbach et al. 2017, Gupta et al. 2017). With this said, policy is operationalized in different ways and across different domains. Socio-cultural norms, behaviour, and attitudes are equally driving influences, alongside the expectations of communities for whom it is intended to serve or impact. But crucially, this requires recognition that this can never result in a ‘one-size-fits-all’ response as people hold preferences for terminology that reflect how they identify as (non)gendered subjects. The ways in which inclusive language is operationalized into different settings requires tailoring, which balances individual patient and community perspectives with the need to be medically accurate. This is because the utility of the word ‘cervix’ is limited by people’s cultural knowledges, literacies, and linguistic preferences, which suggests that the term ‘people with a cervix’ may, at worse, alienate some people from engaging in cervical screening as it may not reflect their deep investment in the identity of ‘woman’ (Ellis 2025). This reflects a more nuanced approach to identifying enablers and barriers to inclusive language in health promotion’s operationalization.

In our previous analyses, we considered the role of policy ecologies to identify how ‘black letter’ policy formalization (see Lea 2020) represents a written form of language that has a flow on to other modalities (Drysdale et al. 2024). Lea (2024) formulates ‘policy ecology’ as both a concept and framework to consider how policies extend beyond their ‘artefactual’ or written form to recognize policy’s ambient and hauntological modalities. This means that policy goes beyond its mandate for use to include the historical and everyday intersections with policies’ pasts, presents, and futures. Policy affects and influences can be seen in shaping everyday conditions whereby policies are tacitly embedded in various contexts and practices, (such as ‘women’s health’), as well as how past policies’ reverberations shape prior social configurations so as they appear predetermined and inevitable (such as cervical screening as something that is provided to women). As such, the expansive ways in which specialist healthcare is consolidated under the umbrella domain of ‘women’s health’, as well as the historical legacies of feminist activism and advocacy in the development of such domains, require recognition of the ambient and hauntological influences. Put simply, the operationalization of inclusive language in health promotion policy facilitates a greater understanding of policy interdependencies and presences, rather than simple measures of success (e.g. the uptake of cervical screening).

It is also important to recognize that such imperatives towards inclusive language are often made through demands to ‘women’s health’ to be more inclusive of diversity, while domains of medical specialities concerning (more implicitly) ‘men’s health’ have received less attention. Yet, by combining this insight to those provided by research on evidence-based practices in mental health policy (Raghavan et al. 2008, Wortham et al. 2023), we highlighted the intersectional and intersectoral relationships between inclusive language in health policy and its downstream operationalization in health promotion. Policies directed towards clinicians only—and thus translated through clinical guidelines and protocols alone—is unlikely to be successful and sustainable (Raghavan et al. 2008, Wortham et al. 2023).

These insights, and the processes of adaptation, also aligns with the Ottawa Charter, which urges action from governments, health and development workers, and communities to participate in the planning and implementation of health care (World Health Organization 1986). The Charter emphasizes that successful health promotion activities involve individual-to-policy-level actions conducted in various settings and with different target populations. As such, health policies can serve as a bridge between research and practice (Lea 2020), and between health programs and their implementation (Bradley et al. 2024). Yet, an available policy or a component within it (i.e. inclusivity in language within Australia’s cervical screening promotion strategy) may not guarantee it is operationalization in practice (Baum and Fisher 2014, Chriqui et al. 2023) if these diffuse processes and practices are not considered.

## CONCLUSION

Inclusive language in cervical screening health promotion policy requires tailoring based on the targeting of communities who may share characteristics that are grouped and defined as currently underscreening populations. But to be effective, health promotion needs to grapple with the relationships and interdependencies between population, community, and individual/patient levels, which often surfaces through pre-existing connections and requires trust. Equally, consideration of the ambient and hautological modalities of health policy requires attention to the historical legacies and everyday meanings associated with women’s health. These findings hold wider implications for how the historical legacies of and contemporary need for ‘women’s health’ can be maintained and respected amid demands for greater gender inclusion. At the same time, the failure to trace diverse and diffuse modes and contexts of operationalization may (re)produce health inequities in practice if left unexamined.

## Supplementary Material

daaf058_suppl_Supplementary_Material

## Data Availability

As this study used qualitative interview methods, there is a potential for re-identification of research participants, the underlying data cannot be made available.
