# Supplementary material for: Beyond words: operationalizing inclusive language in Australian cervical screening health promotion policy
Source: Health Promot Int. 2025 May 20;40(3):daaf058. doi: 10.1093/heapro/daaf058 (PMC12090894; doi:10.1093/heapro/daaf058)
Supplement: daaf058_suppl_Supplementary_Material [file daaf058_suppl_supplementary_material.docx]

**Interview guide: Indicative Questions**

**PRELIMINARIES**

The aim of this research project is explore the role of language in health promotion and practice through a case study of cervical screening. We want to examine what the implications of shift in health promotion language are and thinking about the impact of this on specific populations including but not only those currently identified as being under-screened *[refer to stakeholders area if required: Aboriginal and Torres Strait Islanders, Culturally and Linguistically Diverse populations and Lesbian, Bisexual, Transgender, Queer and Non-binary people].* We are seeking your views to understand decision-making around inclusive language, including gendered and non-gendered language *[explain if necessary*], in the promotion of cervical screening.

*[Verbal consent process if required].*

***Start recording:***

**SECTION ONE: Demographics**

1. Can you tell me a bit about your current role, and your organisation?

(*Prompt: Type of role (policy, service, etc); length of time in current role; professional background and training; populations serviced/targeted; institutional/community/sector service)*

1. What is your/your organisation’s work in connection with Australian National Cervical Screening Program (NCSP)?

*(Prompt: do you/your organisation provide cervical screening as a clinical service, do you promote cervical screening in any communication/material, create educational content for health professionals? Are you involved in supporting the delivery of the NCSP more broadly in the health system?*

1. Have you had any involvement in considering the role of language in general healthcare or cervical screening?

*(Prompt: committee or advisory membership, guideline development, self-collection implementation group, creating copy, etc.)*

**SECTION TWO: Language understanding**

Thinking about how we have historically and commonly referred to cervical cancer as something we should screen for as ‘women’, and thinking about some of the language issues currently at play, what is your take? [explain further if necessary].

1. What have been /are the implications of language for your/your organisation’s work; to what extent is language seen as important, how and to whom?
2. To what extent should language reflect those used in:
   1. Target communities [reflecting cultural change]?
   2. Clinical practice [reflecting medical accuracy?]
   3. Guidelines and other policy/process? [reflecting black letter/formalisation of terms]
3. How important is it that health promotion reflects language and in what context? [client interactions, communication or promotion material, policy/high level communications]?

**SECTION THREE: Process and change**

Continuing with that last theme [remind if needed; adjust to reflect their understanding]:

1. How have you/your organisation responded to some of these language issues at play? What is your/your organisations response? [if not clearly provided in last section]
2. What approach/es have you and or your organisation developed or implemented to language have you taken; can you give examples?

*Prompt:* What are the circumstances in which you have adjusted your approach to the language that you use in [client interactions, communication or promotion material, policy/high level communications]?

1. When developing or implementing these changes, what types of decisions are made about the type of gendered/non-gendered language that you use, who are the key actors/stakeholders involved in decisions (both in terms of high-level development and front-line delivery)?

*Prompt: What are some of the critical moments in decisions for policy, programs and practice? How are stakeholder interests managed? Whose interests are represented?*

1. From your perspective, what are some of the key things to think about when making decisions about language in health care promotion and practice?

*Prompt: governance issues, stakeholder management, staff acceptability and training, settings for screening, community consultation/acceptability, sustainability*

1. From your perspective, what might be some of the enablers/facilitators in the process of adjusting language and as these changes flow in service delivery?

*Prompt: at the health system level/at the community level/at the individual level*

*Prompt*: *How important is adaptability/flexibility and how can this be put into practice?*

**SECTION FOUR: General principles**

Preamble: Its increasingly recognised that the way that we talk about things and label, name things is important in health, and everyday life [reflect major ideas expressed to date if needed]

1. Reflecting on our conversation so far, what are your thoughts on the importance of language in healthcare service delivery, guidelines and community engagement? What is the role that language [explain as gendered, gender-neutral, inclusive] has had for you? Do you have any thoughts about the growing use of non-gendered or gender-neutral language [explain with example of women vs people with a cervix if needed)?

*Prompt: what does gender neutrality mean in this context, what does inclusivity in language mean in this context, what does diversity mean in this context?*

1. From your perspective, what opportunities do changes in language bring about for your organisation? What are some of the benefits? What are some of the challenges? Who might be included based on these changes? Who might be excluded based on these changes?

*Prompt: difference between health literacy, Prompt: on general population/ on under screened populations: Aboriginal and Torres Strait Islanders (including Brotherboys), Culturally and Linguistically Diverse populations, Lesbian, Bisexual, Transgender, Queer and Non-binary people?*

- 1. [Based on response above] What language would you then recommend and why?

1. Thinking beyond cervical screening, or thinking of cervical screening as an example for other services, how far does inclusive language have to go; where does it extend to and where should it stop? In services such as cervical screening? What about other contexts, populations, and services?

*Prompt: What about other gendered services or services ‘for women’: reproduction, menstruation/menopause, childbirth/midwifery, etc. Implications for ‘women’ health as a coherent ‘field’.*

What do you see as the way forward for the NCSP to ensure meaningful inclusivity? How can this be extended to healthcare more generally?

**END**

1. Is there anything else that we haven’t discussed yet that is important to share about your experiences or views?

***End recording***

**Demographic data collection:**

Sex presumed at birth

Gender

Sexuality

Age decade range?

State

Urban/regional/rural

Primary service/function
